# A data science approach to optimize ADHD assessment with the BRIEF-2 questionnaire

**DOI:** 10.1515/tnsci-2022-0349

**Published:** 2024-10-08

**Authors:** Lucía Caselles-Pina, Paula Serna del Amo, David Aguado, Jorge López-Castromán, Juan de Dios Sanjuán-Antúnez, David Delgado-Gómez

**Affiliations:** Department of Statistics, Universidad Carlos III de Madrid, Getafe, Spain; Department of Social Psychology and Methodology, Universidad Autónoma de Madrid, Madrid, Spain; Global Center of Excellence, Hewlett Packard Enterprise, Madrid, Spain; Department of Psychiatry, Nîmes University Hospital, Nîmes, France; IGF, CNRS-INSERM, University of Montpellier, Montpellier, France; Department of Signal Theory and Communications, Carlos III University, Madrid, Spain; CIBERSAM, Madrid, Spain

**Keywords:** Lasso, feature selection, inattention, hyperactivity, neurodevelopment disorders

## Abstract

Attention deficit hyperactivity disorder (ADHD) is a prevalent neurodevelopmental disorder. A key challenge associated with this condition is achieving an early diagnosis. The current study seeks to anticipate and delineate the assessments offered by both parents and teachers concerning a child’s behavior and overall functioning with the Behavior Rating Inventory of Executive Function-2 (BRIEF-2). Mothers, fathers, and teachers of 59 children diagnosed or in the process of being assessed for ADHD participated in this study. The responses provided by 59 mothers, 59 fathers, and 57 teachers to the BRIEF-2 questionnaire were collected. The performance of various feature selection techniques, including Lasso, decision trees, random forest, extreme gradient boosting, and forward stepwise regression, was evaluated. The results indicate that Lasso stands out as the optimal method for our dataset, striking an ideal balance between accuracy and interpretability. A repeated validation analysis reveals an average positive correlation exceeding 0.5 between the inattention/hyperactivity scores reported by informants (mother, father, or teacher) and the predictions derived from Lasso. This performance is achieved using only approximately 18% of the BRIEF-2 items. These findings underscore the usefulness of variable selection techniques in accurately characterizing a patient’s condition while employing a small subset of assessment items. This efficiency is particularly valuable in time-constrained settings and contributes to improving the comprehension of ADHD.

## 1 Introduction

Attention deficit hyperactivity disorder (ADHD) is one of the psychiatric disorders that most affect children and adolescents, with a 7.2% prevalence worldwide [1]. According to the Diagnostic and Statistical Manual of Mental Disorders DSM-5 [2], ADHD is classified as a neurodevelopmental disorder marked by challenges in sustaining attention and significant levels of hyperactivity and impulsivity [3]. Individuals with ADHD face an increased risk of experiencing school failure, accidents, substance abuse, addictions, comorbidities with other psychological disorders, and a potentially shorter life expectancy, among other difficulties [4,5,6]. In addition, ADHD has a strong impact on the social, familiar, and economic spheres [7,8].

An ongoing challenge in clinical psychology is achieving an early diagnosis of ADHD, as early intervention holds the potential to mitigate its long-term impact [9]. The intricacies of this disorder often lead health professionals to rely on questionnaires/scales, performance tests, and predictors obtained with new technologies as part of their evaluation process. Studies have shown that these predictors provide valuable information about different underlying mental constructs [10,11,12]. Among the scales and questionnaires are the Behavior Rating Inventory of Executive Function-2 (BRIEF-2) [13], the DSM-5 criteria [2], the strengths and weaknesses of ADHD symptoms and normal behavior scale [14], or the ADHD rating scale-IV [15]. Examples of performance-based measures are, for example, the Wisconsin-Card Sorting Test [16], the Stroop Test [17], the Tower of London [18], and the Conners Continuous Performance Test II [19]. Predictors based on new technologies are collected, for instance, through virtual reality [20], video games [21,22,23], or motion recognition cameras [24].

This article focuses on improving the efficiency of scales and questionnaires. Despite their widespread use, scales have several weaknesses. On the one hand, the scores obtained from each item are commonly summarized using simple summation or basic statistical techniques. On the other hand, many of the assessment scales that are currently used are composed of a large number of items, which require a considerable amount of time to be administered. This hampers their usability and can lead to fatigue and reduced motivation among the examinees [25].

In order to overcome these limitations, several studies have proposed the use of data science techniques. These achieve a better use of the available information, resulting in an increase in accuracy. In addition, some of these techniques have the ability to discard non-significant variables, effectively reducing the time required for administration. Among these studies, Bledsoe et al. (2020) employed a combination of support vector machines and forward feature selection to enhance the identification of the combined ADHD subtype using a comprehensive battery of neuropsychological measures [26]. The predictors included the Conners parent rating scales [27,28], the Behavioral Assessment System for Children – 2nd Edition (BASC-2) [29], and the d2 test of attention [30]. The authors reported achieving a perfect classification based on these techniques. A limitation of this work is that there are no standard techniques to show the importance of each of the variables used by the support vector machines.

This study aims to explore the feasibility of obtaining accurate estimates of parent and teacher assessments of ADHD using interpretable data science techniques based on feature selection and applied to the BRIEF-2 items. These analytics tools make it possible to discard those items that do not provide significant information on the dependent variable or are redundant without compromising the accuracy of the evaluation. Additionally, identifying the most informative items for each informant facilitates a comparative analysis of similarities and differences between them.

BRIEF-2 is a widely used tool for assessing executive functions in children and adolescents [31]. The theory of executive function comprises a set of cognitive processes necessary for the control and regulation of behavior [2]. Although inattention and hyperactivity are the cardinal symptoms of ADHD, several studies identify their origins in deficits in executive functions as a consequence of a delay in the maturation of the prefrontal cortex [2,32,33]. In addition, in the clinical setting, deficits in executive functions are frequently present in other disorders, such as autism spectrum disorder, learning disorders, and schizophrenia [34].

This theoretical framework of executive functions has been extensively studied in the field of ADHD. Among the most influential works, Barkley proposed a comprehensive model of ADHD that emphasizes deficits in behavioral inhibition and executive functioning as core components of the disorder [35], or the studies conducted by Pennington and Ozonoff [36] and Klingberg [37] that contributed to the understanding of how executive function deficits affect people with ADHD. Identifying these deficits makes it possible to adapt the child’s environment, both at school and at home, to improve their academic performance and daily functioning. Furthermore, this tool can be used to monitor the effectiveness of interventions, allowing personalizing treatments and fostering collaboration between professionals and families [31]. Notably, no previous literature has employed feature selection techniques with the BRIEF-2 scale.

## 2 Methods

### 2.1 Participants

Parents and teachers of 59 children and adolescents, aged between 6 and 18 years, participated in the study. The inclusion criteria required that the children and adolescents either had a confirmed diagnosis of ADHD or were under evaluation for ADHD by a psychiatrist or a clinical psychologist. The mean age of the children and adolescents was 11.06, and 84% were males.

We collaborated with a non-profit association run by parents of children with ADHD that provided access to an anonymized digital database of evaluations, including age, gender, and questionnaire responses.

### 2.2 Assessment

The diagnostic criteria of DSM-5 for ADHD diagnosis and a standardized psychological questionnaire, the BRIEF-2, were administered. These evaluations were directly filled out by mothers, fathers, and teachers of children with ADHD.

#### 2.2.1 Diagnostic criteria of DSM-5 for assessing ADHD symptoms

The DSM-5 diagnostic criteria for the assessment of ADHD symptoms are a set of 18 criteria that describe the symptoms or characteristics that may be present in ADHD [2]. Diagnostic criteria consist of two sets of items, one for inattention (9 criteria) and the other for hyperactivity/impulsivity (9 criteria). Scoring ranges from 0 to 9 for each set of items. Participants completing this assessment reported the presence of these symptoms within the last 6 months. A diagnosis of ADHD is considered if the individual meets six or more persistent symptoms from the inattention and/or hyperactivity/impulsivity lists in two or more different settings (home, school). Hyperactivity/impulsivity and inattention scores obtained with the DSM-5 will be the dependent variables of this study.

#### 2.2.2 BRIEF-2

The Spanish adaptation of the BRIEF-2 was used [31]. This psychometric questionnaire consists of 63 Likert-type items with 3 frequency options (never, sometimes, frequently). It is individually administered to mothers, fathers, and teachers [31]. BRIEF-2 is a globally recognized and extensively used questionnaire, especially in the context of ADHD. Its efficacy lies in its ability to identify impairments related to executive functions in nine domains: inhibition, self-awareness, flexibility, emotional control, initiative, working memory, planning and organization, task monitoring, and organization of materials [38]. It takes approximately 10 min to complete, and the age range is from 5 to 18 years.

When administered to parents and caregivers of 3,063 typically developed children, alpha coefficients ranging from 0.76 to 0.97 have been reported for the parent forms and alpha coefficients ranging from 0.88 to 0.98 for the teacher form [34]. Jiménez and Lucas-Molina demonstrated that Cronbach’s alpha of this scale ranged from 0.71 to 0.91 when completed by parents and caregivers of primary school children (6–15 years old) [39]. Moreover, several studies with different samples have reported positive correlations between the BRIEF-2 and other similar or closely related instruments such as the CBCL, BASC-2, Conners 3, and ADHD-RS [34].

### 2.3 Data analysis techniques

In this article, a similar approach to that of Mooney et al. is followed, where the selected techniques are interpretable [40]. The techniques used were Lasso regression, Decision Trees, Random Forest, Extreme Gradient Boosting (XGBoost), and Forward Stepwise Regression. A brief description of these five techniques is presented below.

– Lasso (least absolute shrinkage and selection operator). The Lasso algorithm is a regularized classification/regression method that accomplishes variable selection and coefficient shrinkage through an L_1_ penalty on the model coefficients [41]. Coefficient shrinkage aims to avoid overfitting the model. That is, it is intended to prevent the model fits very well to the available training data but performing very poorly on data that were not used to build the model. On the other hand, feature selection facilitates the interpretability of the model as it uses fewer variables. Across various fields, numerous studies have consistently demonstrated that this technique substantially enhances both the accuracy and interpretability of models [42,43,44].
– Decision trees. A decision tree is a predictive model used for both regression and classification [45]. It sequentially splits the data into subgroups so that all or most of the elements of a subgroup belong to the same class (classification) or it has a small sum of squared errors (regression). The algorithm gets its name because the partition has the form of an inverted tree.
– Random forest. Random forest is an algorithm that combines multiple decision trees. It focuses on building decision trees from subsamples randomly drawn from the available observations. In addition, the predictors of these subsamples are also randomly drawn from the original ones. Random forest averages the predictions of all the built decision trees [46]. Breiman proposed a method for estimating the importance of each variable so that only those with positive importance are included in the analysis.
– Extreme gradient boosting. XGBoost is a technique based on decision trees where the trees are created sequentially. Each new tree focuses on correctly predicting the observations that were misclassified in the previous trees. To achieve this, these observations are given a higher weight than the rest of the observations [42].
– Forward stepwise regression. This algorithm selects predictors sequentially and, each time one is added, it checks whether it can discard any of those already selected. It starts with an empty set of features and adds predictors progressively until a stopping criterion is met or all variables are included [47].

Analyses were carried out using R (4.2.3) and the following packages: *glmnet* for Lasso [48], randomForest for random forest [49], *rpart* for decision trees [50], *xgboost* for extreme gradient boosting [51], and *leaps* for forward stepwise regression [52].

Furthermore, we computed Cronbach’s alpha to assess internal validity and the kappa coefficient to estimate interrater reliability. In addition, the difference in performance between classifiers is assessed with a paired *t*-test.

### 2.4 Experimental procedures

Before analyzing the data, the imputation of missing values was carried out using the predictive mean matching method [53]. The percentage of missing values on the whole dataset was less than 1%. No other pre-processing was performed. Regarding the predictive analyses, the dependent variable was the child’s inattention score or the hyperactivity/impulsivity score provided by each of the informants through the DSM-5 diagnostic criteria. We executed two experiments, designed to assess the performance of different classification techniques in predicting those scores.

The first experiment consists of evaluating how the analytical techniques described in the previous section can predict the scores of inattention and hyperactivity from the values of the BRIEF-2 items provided by mothers, fathers, and teachers. Therefore, six possible analyses are conducted for each combination of informant and dependent variable. For each of the six scenarios, repeated validation is carried out (*N* = 100) [54]. In each repetition, the observations are randomly divided into a training set (80%) and an evaluation set (20%). The training set is used to estimate the classifier’s parameters through cross-validation, while the evaluation set is employed to assess their performance on an external dataset. To ensure that the results are not influenced by the variability in the complexity of observations within a single evaluation set, this process is repeated 100 times, and the performance metrics from each repetition are averaged. Specifically, for each repetition, we calculate the mean squared error (MSE) between the predicted values of the evaluation observations and the actual values, the correlation, and the number of variables used by each classifier.


**Ethical approval:** The research related to human use has complied with all the relevant national regulations and institutional policies and, in accordance with the tenets of the Helsinki Declaration, has been approved by the author’s institutional review board or equivalent committee. The study was approved by the Ethics Committee at Carlos III University (protocol code CEI21_02_DELGADO and date of approval: February 23, 2021).
**Informed consent**: Patient consent was waived because the data were obtained from a repository where they were anonymized, and therefore, it was not possible to identify patients.

## 3 Results

### 3.1 Experiment 1

The first experiment aims to compare the accuracy obtained by the five aforementioned algorithms in predicting the DSM-5 scores with the responses to the items of the BRIEF-2 questionnaire. It was observed that the mean (standard deviation) of the DSM-5 inattention scores were 6.83 (2.18), 6.10 (2.73), and 5.82 (2.94) for the mother, father, and teacher, respectively. The DSM-5 hyperactivity/impulsivity scores were 5.49 (3.04), 4.52 (2.86), and 4.96 (3.42).

We performed a repeated hold-out validation (*N* = 100). In each of the single validation, the available data were divided into training (80%) and evaluation (20%). The training data were used by means of a five-fold cross-validation to estimate the value of the parameters of each of the five techniques. Performance was measured in terms of the MSE and the correlation (cor) in the evaluation set. Table 1 shows the results for the six possible scenarios (i.e., mother, father, and teacher for both hyperactivity/impulsivity and inattention) as well as the number of BRIEF-2 variables selected (*N* Vars). This table contains the average values of the MSE, cor, and number of selected items in each of the 100 repetitions. According to a \[{\chi }^{2}]\] test, the distribution of the MSE over the 100 repetitions follows a Gaussian distribution.

**Table 1:** Mean square error (standard deviation), average of the number of items selected (standard deviation), and correlation (standard deviation) obtained by each method

		Hyperactivity/impulsivity			Inattention		
		Mother	Father	Teacher	Mother	Father	Teacher
Lasso	MSE	**3.18 (0.97)**	**5.52 (1.5)**	**7.52 (2.64)**	3.23 (1.07)	4.49 (1.55)	4.42 (2.06)
	*N* Vars	16.62 (8.14)	6.94 (5.06)	6.24 (4.24)	14.27 (4.35)	9.98 (6.74)	13.56 (4.45)
	Cor	0.8 (0.09)	0.58 (0.16)	0.6 (0.17)	0.56 (0.16)	0.65 (0.16)	0.73 (0.13)
Random forest	MSE	3.41 (1.02)	5.88 (1.56)	7.77 (2.3)	**2.99 (1.07)**	**4.11 (1.18)**	**4.35 (1.61)**
	*N* Vars	37.78 (3.73)	31.09 (4.93)	32.92 (5.75)	39.82 (5.25)	35.57 (5.25)	43.25 (4.54)
	Cor	0.82 (0.07)	0.52 (0.16)	0.58 (0.17)	0.61 (0.19)	0.67 (0.14)	0.74 (0.12)
Decision trees	MSE	5.16 (1.47)	7.55 (2.35)	9.94 (3.28)	5.17 (1.71)	5.94 (1.74)	6.72 (2.1)
	*N* Vars	14.87 (5.99)	12.77 (3.88)	12.05 (4.19)	17.17 (6.61)	14.86 (5.64)	13.99 (4.76)
	Cor	0.7 (0.1)	0.41 (0.19)	0.49 (0.18)	0.3 (0.23)	0.53 (0.17)	0.57 (0.17)
Stepwise forward selection	MSE	4.22 (1.63)	6 (2.34)	8.96 (3.33)	4.31 (1.38)	4.68 (2.25)	5.9 (2.42)
	*N* Vars	5.1 (3.36)	3.03 (1.61)	3.23 (1.64)	4.5 (2.38)	3.83 (2.96)	4.73 (2.88)
	Cor	0.74 (0.11)	0.54 (0.19)	0.54 (0.16)	0.46 (0.18)	0.64 (0.17)	0.63 (0.16)
XGBoosting	MSE	3.82 (1.23)	6.06 (1.89)	9.08 (2.42)	3.5 (1.23)	4.79 (1.66)	5.72 (1.87)
	*N* Vars	27.9 (5.99)	21.04 (7.79)	23.15 (10.07)	29.21 (6.82)	23.73 (7.22)	26.43 (5.95)
	Cor	0.78 (0.09)	0.53 (0.17)	0.5 (0.17)	0.54 (0.18)	0.62 (0.16)	0.64 (0.15)

Standard deviations are shown in parentheses.

The bold values indicate the smallest MSE among the five analytic techniques.

#### 3.1.1 Examination of the outcomes with hyperactivity as the dependent variable

When examining the hyperactivity/impulsivity scenario in Table 1, Lasso demonstrates the lowest MSEs in the three settings. In addition, Lasso stands out due to the significantly smaller number of variables selected, making it the technique that offers the best trade-off between accuracy and the number of variables selected. It is also observed that the evaluation made by the teacher is the most difficult to predict. These numbers can be attributed to variances between informants, a topic extensively discussed in the literature [55,56]. The weighted Kappa index between parents for the global assessment, based on the DSM-5 diagnostic criteria, was observed to be 0.36. Between the mother and teacher, it was 0.34, and between the father and teacher, it was 0.23.

**Figure 1:**
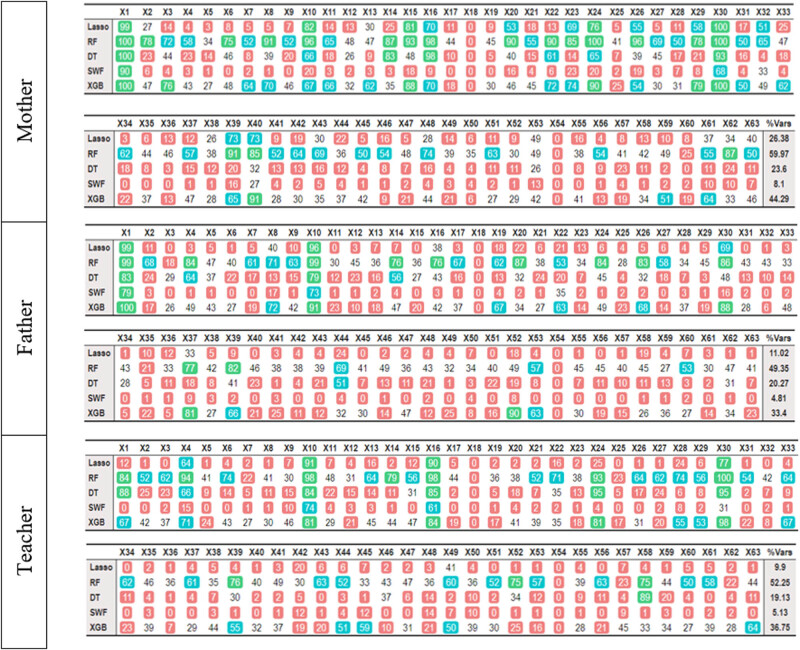
Number of times each variable has been selected in the 100 repetitions when the dependent variable is hyperactivity. The last column (%Vars) shows the proportion of selected features. The cell colors have been established according to the quantiles: red: 0 to 25 times; white: 25 to 50; blue: 50 to 75; and green: 75 to 100.

Figure 1 shows the number of times each variable was selected by each technique.

Independently of the method, the assessment made by the mother is strongly related to the information provided by items 1 (inhibition), 10 (inhibition), 15 (planning), 16 (inhibition), 24 (inhibition), and 30 (inhibition). Except for item 15, the other five items measure hyperactivity. Regarding the father’s evaluation, a smaller number of variables is selected. All five techniques solely consider items 1 (inhibition) and 10 (inhibition) as reliable predictors of the father’s evaluation. Notably, both items 1 and 10 are related to hyperactivity and they were also selected by the mother. Finally, the methods chosen to characterize the teacher’s evaluation, item 10 (which also was selected by the other two informants), and items 16 and 30 (which were selected in the mother’s analysis).

#### 3.1.2 Examination of the outcomes with inattention as the dependent variable

In relation to the performance of techniques in predicting inattention, Random Forest achieved the lowest MSE, as shown in Table 1. However, according to a paired *t*-test, these values do not significantly differ (*p*-value >0.05) from those obtained by Lasso.

**Figure 2:**
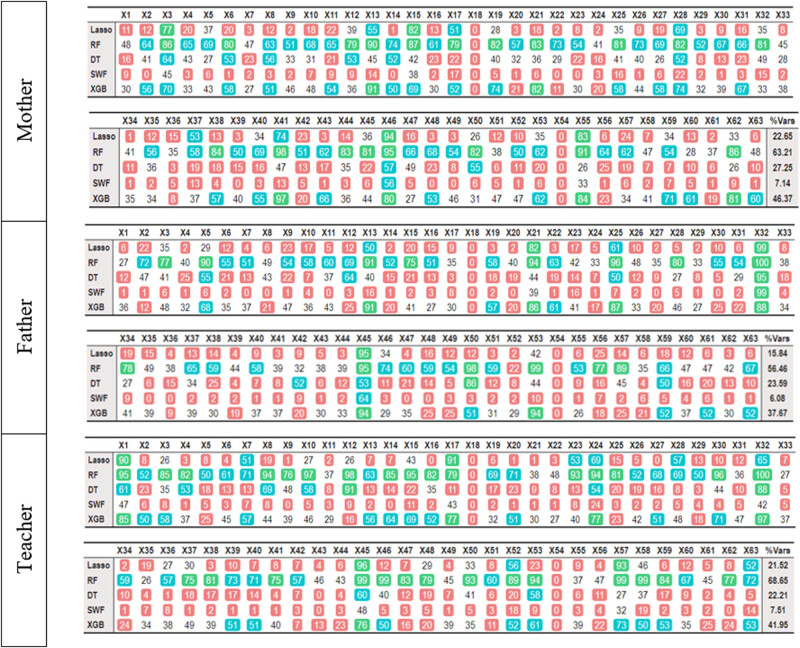
Number of times each variable has been selected in the 100 repetitions when the dependent variable is inattention. The last column (%Vars) shows the proportion of selected features. The cell colors have been established according to the quantiles: red: 0 to 25 times; white: 25 to 50; blue: 50 to 75; and green: 75 to 100.

Based on the assessments provided by different informants, certain items stand out in each case. As shown in Figure 2, when reported by the mother, the focus tends to be on item 3 (working memory), item 28 (working memory), and item 45 (organization of materials). For fathers, the focus is on items 13 (self-awareness), 25 (organization of materials), item 32 (working memory), and item 45 (organization of materials). When the teacher serves as the informant, attention is drawn to item 1 (inhibition), item 24 (inhibition), and item 45 (organization of materials).

The weighted Kappa index between parents for the global assessment, based on the DSM-5 diagnostic criteria, was observed to be 0.32. Between the mother and teacher, it was 0.25, and between the father and teacher, it was 0.07.

### 3.2 Experiment 2

Since the best trade-off between precision and the number of variables was obtained with the Lasso regression, this second experiment aims to understand variable selection in Lasso by considering the importance given to each variable by the informants. The optimal weights for Lasso were determined using all observations and the penalty parameter that minimizes the loss function.

#### 3.2.1 Examination of the outcomes with hyperactivity as the dependent variable

**Figure 3:**
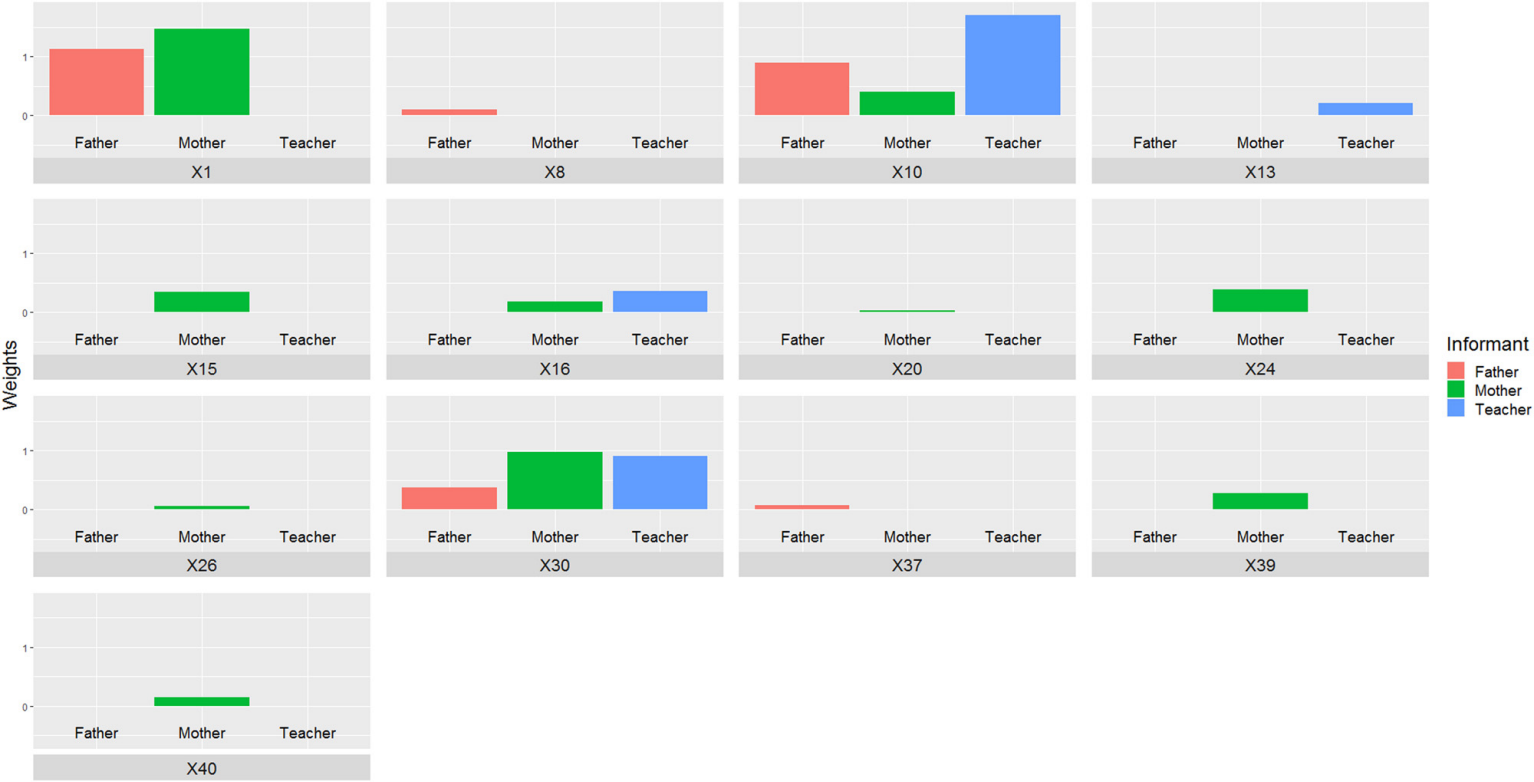
Importance associated with each item by the Lasso method when the dependent variable is hyperactivity.

Figure 3 shows the weights associated with each of the items as a function of the informant when the dependent variable is hyperactivity/impulsivity. As mentioned above, the items selected by all informants are item 10 (inhibition) and item 30 (inhibition). It is also noteworthy to mention item 1 (inhibition), selected by both the father and the mother, which receives large weights.

Finally, it is observed that Lasso also selects items 15 (planning), 16 (inhibition), 20 (self-awareness), 24 (inhibition), 26 (self-awareness), 39 (inhibition), and 40 (planning) filled in by the mother, and to item 8 (organization of materials) and 37 (organization of materials) when filled in by the father. The teacher adds items 13 (self-awareness) and 16 (inhibition) to the three items mentioned above. Furthermore, Cronbach’s alpha was found to be 0.87, 0.72, and 0.81 for the subscales generated when the respondent was the mother, the father, and the teacher, respectively. These values are in line with those documented in the aforementioned literature.

#### 3.2.2 Examination of the outcomes with inattention as the dependent variable

**Figure 4:**
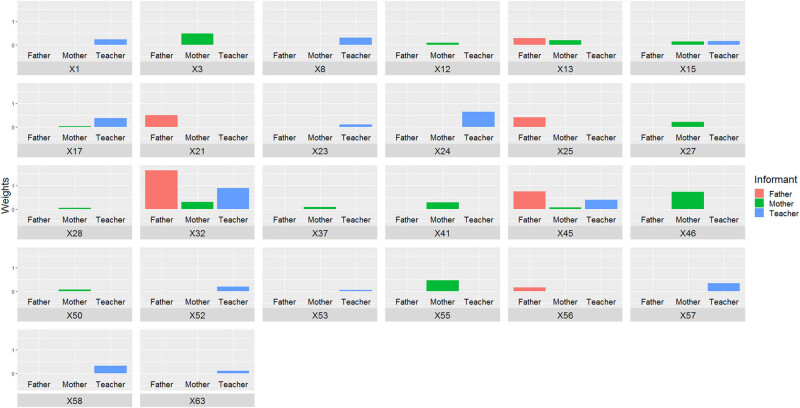
Importance associated with each item by the Lasso method when the dependent variable is inattention.

In this case, the items selected by all informants are item 32 (working memory) and item 45 (organization of materials) (Figure 4). Mothers and teachers agree on items 15 (planning) and 17 (flexibility). Agreement was also observed between fathers and mothers on item 13 (self-awareness).

The next step involves analyzing the other variables selected by each informant. For the mother, the selected items are 3 (working memory), 12 (working memory), 13 (self-awareness), 27 (emotional control), 28 (working memory), 37 (organization of materials), 41 (working memory), 46 (working memory), 50 (initiative), and 55 (initiative). Moving on to the father’s selections, we observe item 13 (self-awareness), item 21 (planning), item 25 (working memory), and item 56 (emotional regulation). Finally, the teacher’s chosen variables include items 1 (inhibition), 8 (organization of materials), 23 (planning), 24 (inhibition), 52 (planning), 53 (planning), 57 (planning), 58 (planning), and 63 (flexibility).

## 4 Discussion

This study focuses on enhancing the assessment of ADHD, based on DSM-5 diagnostic criteria, using the BRIEF-2 scale, which measures the executive functions most affected in ADHD. The study explored various variable selection techniques. The Random Forest technique demonstrated good performance in predicting informant-provided scores for the DSM-5 questionnaire. However, taking into account the six setups, it selected an average of approximately 58% of the available items among informants. On the other hand, Lasso exhibited a better trade-off between the number of variables selected and the MSE, as it selected only an average of 18%.

The characterization of the mothers’ assessment was relatively straightforward for the methods employed, while it proved more challenging in the case of the teachers, as evidenced by the MSE results.

It has been shown that mothers are more likely to identify children with symptomatic ADHD than fathers [57,58]. Mothers and fathers may report different behaviors in the child with ADHD due to variability in home environments. For instance, one parent may spend more time with the child doing outdoor activities, while the other parent may spend more time helping the child plan tasks and activities [58]. The ability of mothers to comprehend and effectively manage their children’s behavior, coupled with their level of knowledge about ADHD, appears to exhibit a noteworthy correlation with the diagnosis of ADHD [59]. Mothers also tend to assign more severe scores. This heightened severity may stem from increased anxiety and depressive symptoms, or it could be linked to more negative mother-child relationships in the context of children with ADHD [60,61,62]. These factors could lead to a higher level of consistency in the responses of mothers, thereby benefiting the prediction model as the frequency and intensity of symptoms become more evident and consistent.

Results have also shown that the error in the teachers’ estimate is greater than that of the parents. In addition, the teachers’ estimates have a larger standard deviation, a consequence of the fact that the scores reported by the teachers show greater variability. This disparity between parent and teacher responses has been highlighted in several studies, such as the recent study by Andersen et al., also using the BRIEF-2 scale [63]. This difference in the way teachers score may be due to several factors. Probably, the scores are influenced by the context. Children do not perform the same at home as they do at school, where they have to be more focused, follow instructions, and interact with their peers [64]. Moreover, in this context, teachers have to interact with many students, which may affect their ability to consistently assess a single child. It may also be due to the influence of teachers’ gender and age/experience. For example, previous studies indicate that female and younger teachers tend to assign harsher scores compared to their older male counterparts [65]. In addition, teachers tend to perceive the behavior of children with ADHD within narrow age ranges and specific contexts [66], whereas parents observe more significant variations in their children’s behaviors, both at home and in diverse settings [67].

An interesting observation regarding the informants, particularly the teachers, is that across all five methods, they consistently selected a greater number of variables from the domain of inattention compared to the domain of hyperactivity/impulsivity. This phenomenon could be attributed to the strong correlation between attention and school performance, making inattention-related behaviors more noticeable and relevant in the eyes of the teachers [68].

The lack of significant equivalence in variable selection between inattention and hyperactivity/impulsivity is unsurprising, given the marked differences in symptoms observed in these two subtypes of ADHD, as outlined in the DSM-5. The only variables selected for both domains of the disorder, hyperactivity/impulsivity and inattention, were items 1 (inhibition), 8 (materials organization), 13 (self-awareness), 15 (planning), 17 (flexibility), 24 (inhibition), and 37 (organization of materials). Except for items 17 and 24, none of the other items were selected by the same informant. Of particular interest is the striking finding that items 10 (inhibition), item 30 (inhibition), 32 (working memory), and item 45 (organization of materials) as highly significant for all informants. Of particular interest is the striking finding that items 1, 10, and 30 (all measuring inhibition) emerge as highly significant, with the majority of techniques selecting this item in over 75% of the 100 conducted iterations. This finding is related to Barkley’s theory, in which inhibition is related to deficits in the executive functions of working memory, self-regulation, internalization of speech, and reconstitution (planning and problem-solving) [35].

Based on the selected items, it is observed that the methods take working memory into account when predicting the assessment of inattention. This finding is related to Baddeley’s working memory model, which describes working memory as a limited capacity system for temporary storage and manipulation of information. The system is composed of four components: the phonological loop that processes and stores verbal and auditory information; the visuospatial sketchpad that processes and stores visual and spatial information; the central executive that coordinates the activities of the two previous systems and manages executive functions; and the episodic buffer that integrates other sources of information [69,70]. Inattention may be related to the deficient functioning of the central executive, who is responsible for directing and maintaining attention. The results are also related to deficits in the supervisory attentional system of the Shallice and Burgess model since Lasso selects several items intended to measure planning along with the items related to working memory [71].

Regarding the hyperactivity/impulsivity dimension, the selected items are very consistent. The algorithm gives the highest weights to items 1, 10, and 30, which measure inhibition. In addition, it also gives high weights to items 16 and 24, which also assess inhibition. The rest of the selected items measure the organization of materials and self-awareness but receive much lower weights.

Before ending the discussion, it might be interesting to mention that there is a reduced 12-item version of the BRIEF-2 [13]. The items of the reduced 12-item scale are related to ADHD since they assess executive functioning as they were obtained from the BRIEF-2 scale. There are numerous studies associating deficits in attention, mood changes, burnout, poor planning, and incomplete tasks with ADHD. However, this short scale was not originally intended to assess ADHD but rather a quick assessment of executive function deficits, leading to the exclusion of items that, according to Lasso, could be more informative. In addition, Lasso differentiates between items that are suitable for assessing inattention and those that are suitable for measuring hyperactivity/impulsivity, and the reduced 12-item scale does not make this distinction.

## 5 Strengths and limitations

This study presents significant strengths, notably the utilization of multiple algorithms to identify the best fit for the dataset and the adoption of an efficient algorithm, specifically Lasso regression. However, our study has a few limitations and should be taken as a preliminary step in the development of a reduced BRIEF-2 scale.

The main limitation is the small sample size, which hinders the possibility of making assumptions about the findings of this study. However, note that the literature has put forth various techniques to address this concern [72], and several studies in the field of ADHD with similar sample sizes have been conducted in this context [73,74,75,76]. Also, children were recruited from an ADHD association, which may imply a selection bias leading to the exclusion of participants with more severe scores. Another limitation is that with the use of Lasso regression, the results may vary depending on the population. Therefore, generalization of the results should be done with caution. Finally, this study lacks the incorporation of clinical variables that could potentially enhance the accuracy of ADHD assessment, such as treatment status or time since symptom onset.

## 6 Conclusion

Given the negative individual and family consequences of ADHD, early detection of this disorder is of utmost importance. For this reason, this article has explored the possibility of optimizing the assessment of ADHD symptoms through machine learning. The results demonstrate that the proposed algorithms, Lasso and Random Forest, have significantly enhanced the accuracy of BRIEF-2 in the assessment of ADHD. Among the two methods, Lasso appears to be the most suitable option due to its ability to select a reduced number of items while still maintaining effectiveness. This finding suggests that Lasso holds promise as a fast and accurate tool for future studies aimed at assisting clinicians in the diagnosis of ADHD. Among the clinical implications of this study is that practitioners can more efficiently manage the time available for consultation as they avoid having to administer non-informative or redundant items. In addition, the present study identifies the most relevant items as those that receive the highest weighting by Lasso. This is important since questionnaires are often summarized as the sum of the scores of their individual items. This assumes that all items are equally informative when, in general, this is not true. If the study conducted in this article is replicated with a larger number of scales and participants, it is possible to obtain a scale with the potential to accurately identify ADHD.
